# On the Extraction of Foreign Bodies from the Esophagus

**Published:** 1850-03

**Authors:** 


*On the Extraction of Foreign Bodies from the Esophagus.* By Professor Dieffenbach.—Foreign bodies observe in their transit certain *stations* at which they halt; thus, in the pharynx behind the thyroid and crycoid cartilages, in the beginning of the gullet, or at its lower end, close to the diaphragm or cardia. They seldom stop at the middle of the gullet. If very large, they may cause suffocation—thus a large piece of meat, a hard boiled egg, a pear, a chestnut, have each proved fatal. Guattani witnessed the most frightful death ensue from a chestnut; the part of the gullet at which it stuck was gangrenous. Spiritus saw the same result follow the swallowing of a five franc piece, which perforated the gullet above the cardiac orifice. Needles, inadvertently swallowed, pierce sometimes the gullet or stomach, advance by the aid of suppuration or otherwise towards the suiface, and either escape spontaneously or through incision. Lyson observed a case where three needles that went in at the mouth came out at the shoulder; I have known one issue at the arm.

The procedure must be modified according to the nature of the substance. None but a bungler would attempt to disgorge a piece of meat sticking at the cardiac opening, or urge on a fragment of glass from the gullet into the stomach. External pressure will suffice for potatoes or plums when stuck in the throat.

For the withdrawal of needles, fish bones, and the like, there is no better implement than a large goose or swan quill feather, with the barbed portion ruffled and imbued with oil. The patient sits with his head leaning upon the breast of an assistant, while the surgeon lowers the tongue, then introduces the feather, with its concave side downwards into the throat, turns it rapidly round, and draws it out. The popular practice of swallowing a crust of bread is sometimes availing, but may also increase the peril when arrested above the bone. A sudden slap on the back is by no means a bad plan when the substance is large and obtuse. It is preferable to that of setting the patient on his head, as was done in the instance of Mr. Brunel to promote the expulsion of the half-sovereign piece.

The principal instruments employed for the present purpose are of the description of forceps. Dupuytren advises, as a preliminary step, the introduction of a gum elastic tube, surmounted with a silver ball, in order to ascertain the position of the foreign body. This, however, is superfluous, and will tend, moreover, to augment irritation. Cooper recommends the forceps of Weiss. The so-called leaden hammer of earlier writers consisted of a lead ball attached to a string, which was let down the throat and pulled up again.

Mesnier's lead hammer was of an olive shape: Petit's was equipped with a wire instead of a string. Petit used besides a metal noose fastened to a whalebone stem; Fabricus Hildanus a many-holed silver tube provided below with a sponge. The double ring of Graefe attached to the end of a rod of whalebone with a steel spring, is very convenient for taking pieces of money out of the throat. The customary instrument, termed repoussoir, or probang, namely, a bit of sponge, as big as a walnut, stuck to the end of a whalebone, is generally useful, either for entangling fish bones and the like, or propeling large round substances. My own procedure is as follows: if the body be small and sharp, I employ the oil feather above described. An oiled wax taper, passed down to the cardiac orifice, has proved serviceable; for, as soon as withdrawn, the body has been rejected. If the body be large, as a portion of flesh meat adherent to a frrgment of bone, I use a lithotrite with an imperforate scoop, and rather straight. The instrument is introduced with the blades closed, until it arrives at its destination, when these are to be separated sufficieutly to grasp the substance, and after a few gentle turns, withdrawn.

When there is impending suffocation from the presence of the very large bodies impacted in the throat, Habicot enjoins tracheotomy before resorting to opening the esophagus, I have never been compelled to this extreme measure. The most difficult thing to deal with are sets of false teeth when swallowed. I once relieved an old lady in this predicament by means of my fingers. On several occasions I have removed, with curved polypus forceps from three to four teeth attached to a gold plate, and which got accidentally all into the throat; once, by the aid of an emetic, as a last resort, a set of four teeth very deeply located.

In all these operations, the patient is to be in a sitting posture; the head properly supported; the mouth rinsed with tepid water; tepid water, mixed with white of egg, taken as a drink, and the instrument smeared with white of egg rather than with oil.—*Med. Times, July* 28, 1849, *p.* 78.

